# Social and Emotional Learning and Academic Achievement in Portuguese Schools: A Bibliometric Study

**DOI:** 10.3389/fpsyg.2017.01913

**Published:** 2017-11-08

**Authors:** Ana M. Cristóvão, Adelinda A. Candeias, José Verdasca

**Affiliations:** ^1^Research Center in Education and Psychology, University of Évora, Évora, Portugal; ^2^Department of Psychology, School of Social Sciences, University of Évora, Évora, Portugal; ^3^Department of Pedagogy and Education, School of Social Sciences, University of Évora, Évora, Portugal

**Keywords:** SEL programs, academic achievement, Portuguese schools, bibliometric study

## Abstract

Social and Emotional Learning (SEL) is an educational movement that is gaining ground throughout the world. We can define SEL as the capacity to recognize and manage emotions, solve problems effectively, and establish positive relationships with others. Research has demonstrated the significant role of SEL in promoting healthy student development and academic achievement. Extensive research confirms that SEL competencies: can be taught, that they promote positive development and reduce problem behaviors, and that they improve students' academic achievement and citizenship. At the international level, several rigorous studies have identified programs and practices that promote SEL. In Portugal, however, no review has yet been published regarding the implementation of SEL programs. Such a study would elucidate the current panorama of SEL programs in Portugal. This study aims to identify research on SEL programs implemented in Portuguese schools and the relationship of those programs with academic achievement. To this end, we have consulted the following databases: Scientific Repository of Open Access of Portugal (RCAAP), Online Knowledge Library (b-on), and Web of Science (WoS). The criteria were: (a) all time frames; (b) publications in either Portuguese or English; (c) programs that developed socio-emotional competencies in Portuguese schools; (d) academic levels including elementary, middle, and high school and (e) students of regular education. Few publications on SEL programs implemented in Portugal were found, although the recent decade has witnessed an upsurge of interest in the topic, principally that arising from academic research.

## Introduction

The twenty-first century challenge for educators, families, and community members is seeking to raise and educate children who are knowledgeable, responsible, caring, and socially competent. A key challenge for schools involves serving culturally diverse students with varied abilities and motivations for learning (Learning First Alliance, 2001). Times have changed. A few generations ago most children would spend only a few years in school. Schools' priority was teaching the traditional subjects, such as reading, writing, and arithmetic. Young people are now spending more years in school and are more exposed to issues of depression, social isolation, or other problems. This requires that they develop concentration, impulse control, and emotional regulation (Lopes and Salovey, 2004).

In the past two decades, research on educational outcomes has demonstrated the importance of positive social behaviors in fostering academic achievement (Blake et al., 2015). According to Greenberg et al. (2003), educators, parents, students, and other members of the educational community believe that today's school has to teach beyond basic skills (reading, writing, counting). Today's school must enhance students' social-emotional competence, character, health, and civic engagement (Greenberg et al., 2003, p. 466).

According to the World Economic Forum's Future of Jobs Report (World Economic Forum, 2016), emotional intelligence will be one of the top 10 job skills in 2020. The awareness that emotional intelligence is an important job skill, in some cases even surpassing technical ability, has been growing in recent years. In any case, we have come to the conclusion that the new generation of children needs something more. Every individual counts, and we will be doing a disservice to the children if we do not orient our educational practices to the individuality of each one.

Awareness of the importance of mental health has been growing worldwide. In Portugal, according to the report citing the First National Epidemiological Study of Mental Health published in 2013, psychiatric disturbances affect more than one fifth of the Portuguese population (Directorate-General for Health, 2013). Common mental disorders (such as depression) are one of the main causes of disability, as expressed, for example, by the high number of deaths and early retirements. According to the Portuguese Directorate-General of Health, common mental disorders are highly prevalent (annually 22.9%, 42.7% throughout life). At the same time, Portugal has been the largest European consumer of benzodiazepines for years, with significant numbers of users of antidepressants and alcoholic beverages. Investigations reveal that some people who develop mental illness in adulthood have manifested signs or had critical episodes during childhood. Some mental illnesses cannot be avoided, but their impact can still be reduced and quality of life can be increased (Directorate-General of Health, 2017).

The National Coordination for Mental Health (2008) has made it a priority to implement mental health education programs including those to promote personal and social skills (Pinto and Raimundo, 2016). Behavioral, emotional, and mental health problems of school children and young people have been growing, significantly impacting school performance, obesity, and risk behaviors (Greenberg et al., 2003; Durlak et al., 2011). The mission of today's school must be reinvented in order to address these problems. In addition to its role in learning and academic performance, the school must actively participate in promoting students' lifelong mental health and well-being (Kickbush, 2012).

Social and Emotional Learning (SEL) is an educational movement gaining ground throughout the world. SEL can be defined as the capacity to recognize and manage emotions, solve problems effectively, and establish positive relationships with others (CASEL, 2003).

A study of the publications about SEL programs implemented in Portuguese schools should provide better understanding of the national panorama regarding the development of social and emotional skills. To this end, we carried out a bibliometric study. According to Archambault et al. (2009), the increased availability of statistics like bibliographic impact makes it increasingly important to understand how publication and citation activities can be included as part of a more holistic review of the literature. Zupic and Cater (2015) pointed out that bibliometric methods employ a quantitative approach for the description, evaluation, and monitoring of published research. They argue that these methods have the potential to introduce a systematic, transparent, and reproducible review process and thus improve the quality of reviews (Zupic and Cater, 2015, p. 430). According to Cortez (2011), the most relevant types of publications are books, theses, chapters of books, articles published in scientific journals, communications in conference proceedings, technical reports, pedagogical materials, white papers, and web pages. Each of these may or may not be subject to peer-review, and they may be national or international in scope (2011, p. 3).

Bibliometric studies enable us to identify published studies on SEL implementation in Portuguese schools, while also informing us about the scientific activity of researchers and universities.

## Social and emotional learning—SEL

Emotions can facilitate or impede children's academic engagement, commitment, and ultimate school success since relationships and emotional processes affect how and what we learn. Thus, schools and families must effectively address these aspects of the educational process for the benefit of all students (Elias et al., 1997).

In 1994 a group of professors, researchers and health care professionals held a meeting at the Fetzer Institute to reflect on how to improve students' social and emotional competences and school performance. The concept of SEL emerged from this meeting. It can be defined as a strategy to nurture students' social and emotional competences by way of explicit teaching. SEL uses a student-centered approach that encourages student participation in the learning process and in the development of analytical communication and collaborative behaviors (CASEL, 2012; Weissberg et al., 2015).

These participants created the Collaborative for Academic, Social, and Emotional Learning (CASEL), a nonprofit organization in Chicago which has been at the forefront of North American and international efforts to promote SEL. Founded by Daniel Goleman and Eileen Growald, CASEL's mission is to establish SEL as an essential part of education. “We envision the world where families, schools, and communities work together to support the healthy development of all children. All children will become engaged lifelong learners who are self-aware, who are caring and connected to others, and who make responsible decisions” (CASEL, 2003, p. 5). CASEL defines SEL as the process by which people develop their social and emotional competencies for “success in school and in the workplace, including the skills necessary to recognize and manage emotions, develop care, and concern for others, form positive relationships, make responsible decisions, and successfully handle the demands of growing up in today's complex society.” (CASEL, 2012, p. 4). One of the most commonly used definitions is that of Elias et al. (1997). They see SEL as a process through which we learn to recognize and manage emotions, care about others, make good decisions, behave ethically and responsibly, develop positive relationships, and avoid negative behaviors.

The SEL approach defends that, as with academic skills, the development of social and emotional competencies must be accomplished through explicit instruction. According to Weissberg et al. (2015) one of the most prevalent SEL approaches “involves training teachers to deliver explicit lessons that teach social and emotional skills, then finding opportunities for students to reinforce their use throughout the day” (2015, p. 8). The development of social and emotional competences in the SEL approach occurs within and outside the classroom in a school context, but also at the family, community, and political levels (Weissberg et al., 2015). Teachers must be the engine that drives SEL programs. Recently, Schonert-Reicht Kimberly (2017) examined the role of teachers in implementing SEL programs and practices in schools and classrooms. The author concludes the success of SEL programs is directly related to teachers' beliefs and their well-being. Hence the importance of teacher training in SEL and the importance of explicitly promoting SEL in initial teacher training (Schonert-Reicht Kimberly, 2017).

SEL programming is based on understanding that many different kinds of problem behaviors are caused by the same or similar risk factors, and the best learning emerges from supportive relationships that make learning both challenging and meaningful (CASEL, 2003).

CASEL recommends that SEL Programs should have as direct targets five key competencies: (1) Competence in self-awareness—the ability to understand one's own emotions, personal goals, and values. This includes accurately assessing one's strengths and limitations and possessing a well-grounded sense of confidence and optimism. (2) Competencies in self-management—the ability to regulate emotions and behaviors. This includes managing stress, controlling impulses, and setting and working toward achieving personal and academic goals. (3) Competence in social awareness—the ability to take the perspective of those from different cultures and backgrounds. (4) Competence in relationship skills—providing children with the tools they need to establish and maintain healthy and rewarding relationships. (5) Competencies in responsible decision making—the ability to consider ethical standards, safety concerns, and accurate behavioral norms for risky behaviors, to realistically evaluate the consequences of various actions, and to take the health and well-being of self and others into consideration (CASEL, 2003). These five CASEL competencies reflect intrapersonal and interpersonal domains (National Research Council, 2012). Self-awareness and self-management deal with issues within the intrapersonal domain, whereas social awareness and relationship skills are interpersonal. Responsible decision-making is both an individual and social process and therefore overlaps both domains (CASEL, 2012).

According to Brackett et al. (2016) a unified vision for SEL exists that promotes the children's fullest potential (socially, emotionally, and academically). They state that there are many ways to realize this vision “what makes each approach to SEL unique is the specific content it includes, how the presentation of content is sustained with quality within a school organization over time.” (2016, p. 21).

Zins et al. present the essential characteristics of effective SEL programming. They posit that the most effective SEL efforts use comprehensive, multiyear, multicomponent approaches (Zins et al., 2004, p8). Successful programs must be carefully planned, and they must be both theory and research based. Students should be taught to apply SEL skills to daily life. Both the affective and social dimension of learning should be addressed. Initiatives should lead to coordinated, integrated and unified programming linked to academic outcomes. They should address key implementation factors to support effective social and emotional learning and development. Family-community partnerships should be nurtured and the design should include components aimed at continuous improvement, evaluation of outcomes, and dissemination (Zins et al., 2004, p. 10–11). For skills to become part of children's active repertoire, they need to be learned, supported, and, furthermore, they need to be valued in a range of contexts. Elias and colleagues present five main characteristics that contexts must have: (1) a school climate that articulates specific themes and values such as respect, responsibility and honesty, and conveys an overall sense of purpose for attending school; (2) explicit instruction and practice in skills for participatory competence; (3) developmentally appropriate instruction so as to promote health and prevent specific problems; (4) services that enhance students' coping skills and provide social support; and 5) widespread, systematic opportunities for positive contributory service (Elias et al., 2015, p. 35).

There are a large number of correlational and longitudinal studies that indicate that the development of socio-emotional competencies contributes to better psychosocial adjustment of students, and improved attitudes, academic and behavioral results (Weissberg et al., 2015).

Weissberg et al. (2015) conducted a pertinent discussion about the results of the most influential SEL studies. The first was the first comprehensive survey of existing school-based SEL programs, the *Safe and Sound: An Educational Leader's Guide to Evidence-Based Social and Emotional Learning (SEL) Programs* (CASEL, 2003). This publication was very important and became a popular source of information. It provided educators with practical information about procedural aspects and shared the outcomes of several SEL programs. In 2013 CASEL published *Guide: Effective Social and Emotional Learning Programs—Preschool and Elementary School Edition*. It was based on more rigorous research criteria than those used in 2003. This important guide focused on successful preschool and elementary school outcomes. Another important publication was that of Zins et al. (2004) *Building Academic Success on Social and Emotional Learning: What Does the Research Say*. This article reinforced the notion that SEL programs can improve students' success in school and in life. The last work was Durlak et al. (2011). It involved an important, large-scale meta-analysis of studies involving over 270,000 students and confirmed that SEL produces significant positive effects in different aspects of adjustment, including improvements in academic performance, SEL skills, prosocial behaviors, and attitudes toward self and others. In summary, Weissberg et al. concluded that “well-implemented SEL programs are an evidence-based approach that not only improves student's academic, behavioral, and personal adjustment but also prevents some important negative outcomes” (Weissberg et al., 2015, p. 12–13).

In 2015 the *Handbook of Social and Emotional Learning: Research and Practice* edited by Durlak, Domitrovich, Weissberg, and Gullota was published. This comprehensive and definitive handbook is a testimony to the extraordinary SEL work over the last two decades. It covers all aspects of SEL research, practice, and policy. The conceptual and scientific underpinnings of SEL are reviewed and the approach's relationship to children's and adolescents' academic success and mental health is examined. In-depth analyses of SEL implementations and assessments in diverse educational settings are described including the roles of school- and district-level leadership, teacher training, and school-family partnerships. This publication shows there is a large body of scientific evidence demonstrating the positive outcomes of SEL.

### SEL programs and academic achievement

**Figure 1:**
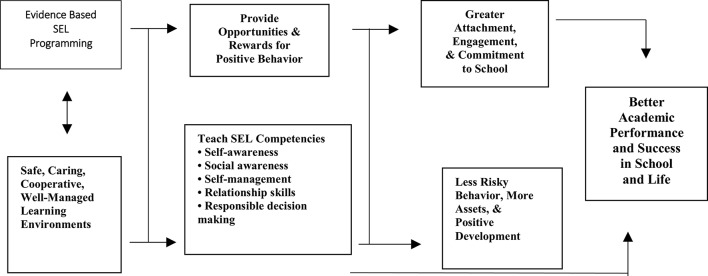
Evidence-based SEL programming paths to success in school and in life by Zins et al. (2004).

Schools will be most successful in their educational mission when they integrate efforts to promote children's academic, social, and emotional learning (Elias et al., 1997). Social and emotional learning has a critical role in improving children's academic performance and lifelong learning. Researchers have demonstrated that SEL plays important roles in influencing nonacademic outcomes, but also has a critical role in improving children's academic performance and lifelong learning (Zins et al., 2004). *Building Academic Success on Social and Emotional Learning* edited by Zins et al. (2004) presents considerable evidence that SEL can not only improve students' social development and mental health but can strengthen their academic achievement. Figure 1 illustrates the connection between evidence-based SEL programming and better academic performance and success in school and in life.

In their words, this figure “indicates that SEL interventions and skill development should occur within a supportive learning environment (…) As a result, opportunities for reward are created and SEL competencies are developed and reinforced (…) The final outcome is improved performance in school and life” (Zins et al., 2004, p. 9). The authors call for consistent studies to demonstrate that socio-emotional skills improve academic performance (2004, p. 193).

Analyses of recent school-based prevention programs provide general agreement that some of these programs are effective in reducing maladaptive behaviors and improving school success (Durlak and Wells, 1997; Durlak et al., 2011). Payton et al. (2008) conducted three large-scale reviews (of 317 studies involving 324,303 children) on the impact of SEL on elementary and middle-school students. The reviews demonstrated gains of 11–17 percentile points on achievement tests that demonstrated that SEL programs offer students a practical educational benefit. Durlak et al. (2011) conducted one meta-analysis of 213 school-based initiatives involving more than 270,000 students who participated in evidence-based SEL programs and it showed an 11 percentile-point gain in academic achievement compared to students who did not participate in SEL programs. These reviews showed improved classroom behavior, an increased ability to manage stress and depression, and better attitudes about themselves, others, and school for students who participated in SEL programs (Payton et al., 2008; Durlak et al., 2011).

CASEL has carried out an extensive body of rigorous research (including randomized control trials, longitudinal follow-ups, and multiple replications) that demonstrates that education that promotes SEL gets results and that teachers in all academic areas can effectively teach SEL (CASEL, 2016).

### SEL programs in Portugal

Most of the SEL studies have taken place in U.S. schools. Torrente et al. (2015) reviewed European SEL policy and found “an extremely diverse panorama in terms of the presence, progress, and origins of SEL and initiatives” (p. 569). They found that much progress is to be made in Portugal “in agreeing on its meaning and practical implications” (Menezes, 2003, p. 572). The first reference to social and emotional education in Portugal emerged only in 2011 (Faria, 2011).

The Foundation Law of the Educational System, Law n° 86/1986, the normative reference for educational policies that aim toward the development of education and the educational system, stipulated in article 50 that “the curricular organization of schools shall take into account, at both the horizontal and vertical levels, a well-balanced promotion of students' physical, motor, cognitive, affective, aesthetic, social, and moral development.” In the 30 years following the 1986 law, Portuguese schools have experienced many reforms, both those instigated by various governments and others, to promote the issues of social and emotional development.

The specific promotion of social and emotional skills only began in the 1990s however, with the emergence of some intervention programs, often driven by local entities or associations. In 1997, the Ministry of Education published a pioneering program to boost social and emotional competencies, the *Manual of the Program of Promotion and Education for Health*. A decade of research on this program presented as principal vectors the importance of teacher training for the program's implementation, the usefulness of promoting these competences in school contexts, and a lack of preventative measures and the scarity of promotion of positive behaviors (Pinto and Raimundo, 2016).

According to Costa and Faria (2013) SEL in Portugal still has a long way to go toward its institutionalization in schools (2013, p. 419). Recently, however, we have witnessed a rising interest in this question by the Portuguese government. In 2016, the Ministry of Health published the *Manual for the Promotion of Social and Emotional Competencies in Schools*. Its main objective was to serve as a pedagogical resource to facilitate the formation and implementation of a project promoting Mental Health in schools based on SEL programs (de Carvalho et al., 2017). To date, no data have been published regarding the implementation and effectiveness of this program.

In February 2017, the Portuguese government commissioned a national reference group to draw up a document entitled the “Profile of students dropping out of compulsory schooling” (Martins et al., 2017) with the following components: (a) a humanistic profile; (b) educating by teaching for practical achievement of learning; (c) making inclusion a requisite of education; (d) contributing to sustainable development; (e) educating by teaching with consistency and flexibility; (f) acting with adaptability and daring; g) guaranteeing stability; and (h) valorizing knowledge (Martins et al., 2017). The student profile in this document is based on a humanist profile which “means focusing on a person-centered society guided by the fundamental value of human dignity” (Martins et al., 2017, p. 6). The document described 10 key competencies that students must have at the end of compulsory schooling, and we highlight those of interpersonal relationships, personal development, and autonomy. Interpersonal relationship competencies relate to “interaction with others, which occurs in different social and emotional contexts. They allow us to recognize, express, and manage emotions, build relationships, set goals and respond to personal and social needs,” (Martins et al., 2017, p. 15).

Personal development and autonomy skills concern the “process by which the student develops his capacity to integrate thought, emotion, and behavior, building self-confidence, motivation to learn, self-regulation, self-initiative, and informed decision-making, which enable a growing autonomy in the various dimensions of knowledge, know-how, know-how, and action” (Martins et al., 2017, p. 15).

A set of teaching initiatives decisive for the development of students' profiles were presented. We highlight: (a) purposefully and systematically encouraging activities inside and outside of the classroom that allow students to make choices, exchange points of view, solve problems and make value based decisions; (b) creating time and space within schools for students to act freely and responsibly; (c) when evaluating student learning, valorizing work arising from students' free initiative, and encouraging their positive actions within the school and the community (Martins et al., 2017, p. 18).

This document can be a lever for the School to develop a broader and holistic student development model that integrates young people's social and emotional development.

It is important to identify investigations that demonstrate the implementation of SEL programs in Portuguese schools and their relationship with school achievement, while also raising awareness within the educational community of good practices within the schools. To this end, we carried out a bibliometric study and a systematic review of the literature, looking for publications that cover the implementation of SEL programs and their relationship to academic performance.

## Methods

There is a need to analyze the effects of SEL programs on academic achievement (Durlak et al., 2011; CASEL, 2013). The lack of effectiveness studies of SEL programs in the Portuguese context was the springboard for this study, whose main purpose was to find SEL programs in Portuguese schools and whether relationships had been established between these programs and academic achievement. The following questions guided the review: (1) How many SEL projects exist in Portuguese schools? (2) Does Portuguese SEL mention the relationship to academic achievement?

To answer these questions we set the following objectives: (a) to identify the number of papers that looked at SEL programs in Portuguese schools; (b) to analyze when more publications took place; (c) to investigate publication type; (d) to evaluate the geographical distribution of the selected documents; (e) to identify Portuguese SEL, in particular, the years of implementation and students involved, and (f) to gathering data about the relationship between SEL programs and academic achievement.

The methodological approach blended technical bibliometric and content analysis. The bibliometric analysis permitted us to identify trends in terms of the number of publications over time, main authors and works (Neely, 2005). Content analysis codified and analyzed the main themes in the literature (Bardin, 2008).

Data analysis was performed in two stages. Firstly, the primary data were treated, seeking to form a quantitative view of the publications, grouping them by year of publication and journal. The title and abstracts were copied to a database using Microsoft Excel 2013.

Content analysis comprised the second stage. The articles were classified regarding the presence or not of data regarding the relationship between SEL programs and academic results.

### Procedure

Three databases were thought to serve our goals: (1) Scientific Repository of Open Access of Portugal (RCAAP); (2) Online Knowledge Library (b-on), and (3) Web of Science (WoS). The Scientific Repository of Open Access of Portugal (RCAAP) database aims to collect, aggregate and index Open Access scientific contents from Portuguese institutional repositories. It is a single entry point for searching, discovering, and recalling thousands of scientific and scholarly publications, namely journal articles, conference papers, thesis, and dissertations, distributed by several Portuguese repositories. “Open Access” in Portugal consists of the free Internet publication of peer-reviewed journals, dissertations, thesis, conference communications and technical reports. According to the stipulated objectives, this database has proved to be the most complete. In the course of the investigation, we also searched the B-on databases and WoS database, as we are interested in knowing what was published internationally. The Online Library of Knowledge (b-on) provides unlimited access to full texts from thousands of scientific journals and online e-books from some of the world's leading content providers. Web of Science is owned by Thomson Reuters, a database that provides access to more than 9,200 journal titles.

The following criteria guided the searches: (a) documents published through 2016; (b) English keywords Social and Emotional Learning; (c) Portuguese keywords with various synonyms (e.g., aprendizagem socioemocional; aprendizagem socio-emocional, aprendizagem social e emocional); (d) only SEL programs implemented in Portuguese schools; (e) school level [e.g., elementary, middle, and high school and (f) students of regular education].

The initial search produced 2,436 documents in the RCAAP, 311 in B-on and 9 in the WoS, resulting in a total of 2,756 documents, of which 556 were repeated, leaving a total of 2,200 works for analysis. Applying the inclusion criteria reduced the list to only 19 publications requiring deeper analysis.

## Results

The following section discusses the results of the analyses conducted from the sample obtained so that it is possible to have an overview of publications related to SEL programs implemented in Portuguese schools and their relation to academic performance.

**Figure 2:**
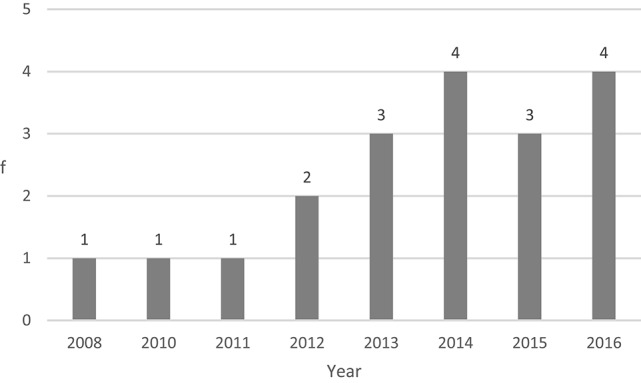
Evolution of publications over time.

In the initial analysis, we observed the number of publications that deal with SEL programs implemented in Portuguese schools. It is worth mentioning that there has been a growth in recent years (Figure 2). As Figure 2 illustrates, no publications dealing with Portugal pre-date 2008, although international SEL programs are over 20 years old. Most investigations have been published in the last 3 years (*n* = 11; 58%). It is worth highlighting how few studies there are in the literature regarding SEL implementation in Portugal.

**Figure 3:**
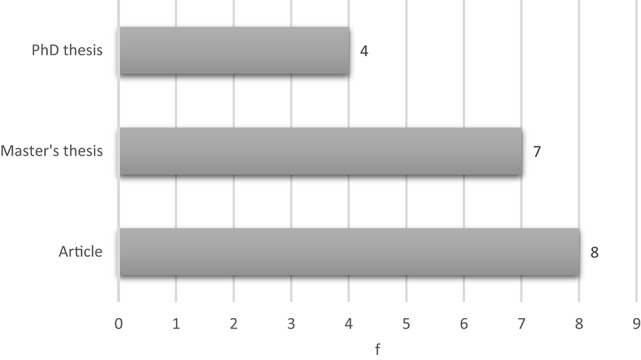
Graph of the type of the publications.

Figure 3 shows the distribution of the research analyzed by the type of publication. Academic research (master's and Ph.D. theses) are most prominent (*n* = 11; 58%), with master's theses predominating (*n* = 7).

**Figure 4:**
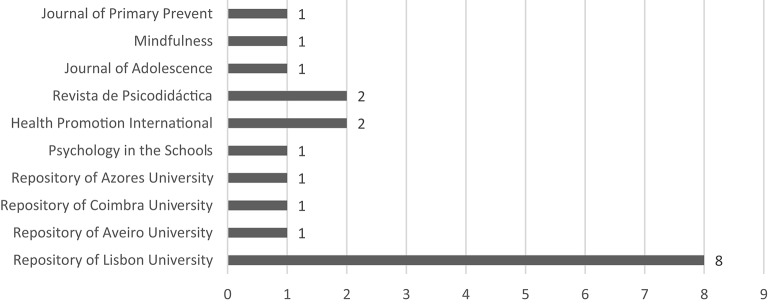
Graph of the origin of the publications.

**Figure 5:**
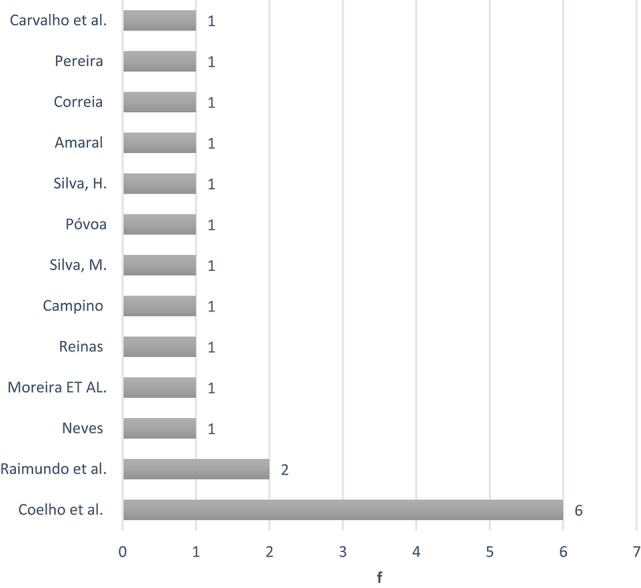
Authors' chart by number of articles published.

That data showed considerable dispersion among different authors and institutional affiliations as illustrated in Figures 4, 5.

Nineteen publications originated from 10 different sources. Figure 4 shows us that the University of Lisbon produced the most publications (*n* = 8) with Vitor Coelho having authored the largest number (Figure 5).

Table 1 illustrates the most often cited articles. Many publications provided no information about citations. Moreira et al. (2010) and Coelho et al. (2014, 2015a,b) were the most frequently cited, with 11 and 8 citations, respectively.

**Table 1:** Most cited articles.

**Author and year of publication**	**Journal**	**Title of article**	**Number of citations**
Moreira et al., 2010	Health Promotion International	Evaluation of a manual-based programme for the promotion of social and emotional skills in elementary school children: results from a 4-year study in Portugal	11
Raimundo et al., 2013	Psychology in the Schools	The effects of a social–emotional learning program on elementary school children: the role of pupils' characteristics	4
Coelho et al., 2014	Revista de Psicodidáctica	The Impact of a School-Based Social and Emotional Learning Program on the Self-Concept of Middle School Students	8
Coelho et al., 2015a	Journal of Adolescence	“Positive Attitude”: A multilevel model analysis of the effectiveness of a Social and Emotional Learning Program for Portuguese middle school students	2
Coelho et al., 2015b	Health Promotion International	The impact of a Portuguese middle school social–emotional learning program	8

Regarding SEL Programs, the “Positive Attitude Program” has the most references (*n* = 6), followed by the “Slowly but steadily” (*n* = 3). It should also be mentioned that all the articles analyzed were published in international journals.

Table 2 illustrates the results of our content analysis.

**Table 2:** SEL intervention programs studies.

**Author and year of publication**	**Name of program**	**Number of students**	**Grade**	**Number of sessions or hours**	**Local**	**Targeted student outcomes: references to academic achievement**
Neves, 2008	The Best of Coping (BOC)	98	Middle School	10 sessions	Lisbon district	*“(…) promotes school performance by working on the affective and social dimensions of academic learning, such as competencies that encourage participation in the classroom (…)”* (p. 163)
Moreira et al., 2010	Growing Up Playing (GUP)	First year 843 Second year: 1,446 Third year: 1,077 Fourth year:560	Elementary schools	10 h	Braga, Lisbon, Viana do Castelo, Aveiro district	Does not refer
Reinas, 2011	Slowly but steadly	213	Elementary school	21 sessions	Lisbon district	Does not refer
Raimundo, 2012	Slowly but steadly	213	Elementary school	21 sessions	Lisbon district	“*A two-way mixed ANOVA 2 × 2 with “group” (intervention vs. control) as a between subjects factor and “time” (post-test vs. follow-up) as a within-subjects factor showed a significant interaction effect with a significant advantage for the intervention group on academic performance (…)”* (p. 104)
Campino, 2012	Program for the promotion of socio-emotional competencies	41	Elementary school (5 grade) and Middle school (6 grade)	18 sessions	Lisbon district	Does not refer
Silva, 2013	International Baccalaureate Diploma Programme (IBDP)	70–90	High school	240 h	Lisbon District	“*(…) influence of social and emotional learning in safe, well-managed, attentive and participatory learning environments, increasing the student's attachment to school, the collaborative work of the entire educational community, interpersonal relationships and pro-social behaviors as conducive to impacts on results academic and in the integral development of the student.”* (p. 114)
Póvoa, 2013	Emotion School	19	Middle schol (7 and 8 grade) and High school (9 grade)	1 session per week (6 months)	Aveiro district	“*(…) contributes significantly not only to a positive change in the behavior and relationships that the students establish with their peers, but also to the improvement of school results”* (p. 44)
Raimundo et al., 2013	Slowly but steadly	213	Elementary school	Weekly sessions (1 year)	Lisbon district	“*The impact on not only behavioral but also academic outcomes should be considered.”* (p. 178)
Silva, 2014	Me & The Others—History 9	58	High school	7 sessions	Lisbon district	Does not refer
Coelho, 2014	Positive Attitude	First study: 970	Elementary school	13 sessions	Lisbon district	“*Particularly important is the finding of a very relevant relationship between participation in school adjustment programs in the transition to the second cycle, accompanied by a program of development of socio-emotional skills, and reduction of absenteeism and school failure in the 5th year.”* (Coelho, 2014, p. 297)
	Let's Feel with Necas	Second study: 472	Middle school	13 sessions		
	Positive Attitude	Third study: 825	Elementary school	20 sessions		
Amaral, 2014	Positive Attitude	139	Elementary school	11 sessions	Azores archipelago	Does not refer
Coelho et al., 2014	Positive Attitude	474	Middle School	13 sessions	Lisbon district	“*In academic self-concept there were significant increases for students in the intervention group in the lowest quartile, even though there no significant increases for the intervention groups over control groups as a whole for the total sample, while for social self-concept the effect size of the intervention was large when for the total sample it was medium.”* (p. 361)
Coelho et al., 2015a	Positive Attitude	855	Middle School	12 sessions	Lisbon district	Does not refer
Coelho et al., 2015b	Positive Attitude	472	Middle School	13 sessions	Lisbon district	Does not refer
Correia, 2015	Live the emotions	First study : 67	Preschool	18 sessions	Lisbon district	“*There were significant gains with intervention, regardless of the previous level of competence and gender, in the relationship between peers, academic behavior, social skills, emotional knowledge, school learning skills, school, behavioral and social adaptation.” (2015)*.
	Positive Attitude	Second Study: 144	Elementary school			
de Carvalho et al., 2017	Positive Attitude	454	Elementary school	6 sessions	Lisbon district	“*In accordance with previous studies, children who participated in the MindUP program improved in positive emotions, which can contribute to flourishing mental health and to improving academic results”*
Pereira, 2016		45	Middle School	9 sessions	Lisbon district	“*First of all, results suggest that SEL programs may help to accomplish the mission of educational systems to deliver effective interventions that meet the academic, behavioral, and socio-emotional needs of children and youth (…)*” (p. 70)
Coelho et al., 2016a	Positive Attitude	2,068	Elementary school and Middle schol	13 sessions	Lisbon district	Does not refer
Coelho et al., 2016b	Positive Attitude	970	Elementary school	13 sessions	Lisbon district	Does not refer

The “Positive Attitude” SEL program of Vitor Coelho et al. involved the most students. Only four of the investigations dealt with fewer than 100 student SEL participants. It is also of interest that most of the SEL Programs were implemented in the district of Lisbon, with only two studies taking place in other locations. Finally, as Table 2 illustrates, most SEL Programs, with only two exceptions, refer to the relationship between programs and academic achievement. Although, the literature indicates that one of the results of the implementation of SEL Programs is the improvement of academic performance, in the studies analyzed only nine studies refer to this relationship.

## Discussion

The discussion around twenty-first century competencies has rekindled the debate about the importance of developing social and emotional competencies in our children and young people. The promotion of social and emotional competencies within the Portuguese educational context has seen advances and retreats. Costa and Faria's (2013) analysis clearly presents the introduction of disciplinary areas at certain times in the last 30 years (example: Project Area, Personal, and Social Development) and their exclusion. They see a preference for performance-oriented education vs. the integration of the personal and social competencies and the search for greater understanding of their contribution to preventing academic failure (Costa and Faria, 2013). They trace these changes to political changes within the Ministry of Education.

School culture must also be referred to, namely teacher resistance to innovative practices that might bring improvements to students and to the school environment. Santo and Alves (2015) affirm that resistance in schools is notorious oftentimes because of the “lack of commitment of the teachers and students, a latent conflict of dissatisfaction that does not allow a cooperative work that leads to the implementation of new practices inside and outside the classroom” (p. 1,061).

We are experiencing a special moment in Portugal, due to the extension of compulsory schooling to 12 years of schooling or 18 years of age, and this has brought various challenges to schools, teachers, and society as a whole. A 2014 study on the enlargement of schools concluded in part that “it is fundamental that the school prepares young people to overcome their difficulties autonomously” (Cid et al., 2014, p. 121).

The challenges of the twenty-first century on the one hand, on the other hand, the challenges of the Portuguese education reforms were the motto for the public discussion of the Profile of students at the end of compulsory schooling. According to Martins et al. (2017), the discussion is based on a “model of schooling oriented toward student learning, which aims at both individual qualification and democratic citizenship” (p. 10). This is an important moment in the Portuguese context that we believe may bring important changes regarding the promotion of citizenship skills in our students.

In conclusion, we defend the SEL approach aimed at developing five key competencies: self-awareness, social awareness, self-control, relational skills, and responsible decision making (CASEL, 2003). In our view, this is a promising approach to supporting students to deal adequately with the demands of today's society while also promoting greater school success.

## Conclusion

This study identified a dearth of literature about the implementation of SEL programs in Portuguese schools. Only 17 publications met our research criteria, seven of which belonged^***^ to a single SEL initiative, the Positive Attitude program. We therefore conclude that, in addition to the lack of research, there also is little diversity among SEL programs. The first publications only began to appear in 2008, although recent years have witnessed an increase. Due to the impact of developing students' socio-emotional competences their behavior and academic success, we argue that this issue should receive greater attention by Portuguese researchers. More investigation should be carried out and published and the flow of publications as well as their quality should be monitored. Other countries, such as the U.S.A. and Spain are much more productive in this respect (Torrente et al., 2015). Our review of the research identified that academic investigations (masters and doctoral theses) comprised the vast majority of the literature on this issue, which suggests that universities play an important role in disseminating the SEL methodology. However, we believe that, in addition to academic researchers, Portuguese schoolteachers should be encouraged to publish what is done within their schools to encourage socio-emotional competences. Knowledge should not be hidden behind school walls.

We concluded from our analysis that research on Portuguese SEL programs and their relationship with academic success is still quite dispersed, both in relation to publication vehicles and in relation to authors and works of reference.

This study further suggests the need for inservice training about this issue as well as the importance of preparing new teachers in SEL (Schonert-Reicht Kimberly, 2017). One study of this type is limited due to the source and availability of data. We faced the fact that our institution did not have access to all of the available data bases, nor were many publications available for consultation.

## Author contributions

Conceptualization: AMC and AAC; Methodology: AMC and JV; Formal Analysis: JV; Investigation: AMC; Writing-Review and Editing: AMC and AAC; Supervision: AMC and JV.

### Conflict of interest statement

The authors declare that the research was conducted in the absence of any commercial or financial relationships that could be construed as a potential conflict of interest.
